# The impact of metabolic plasticity on winter energy use models

**DOI:** 10.1242/jeb.243422

**Published:** 2022-02-25

**Authors:** Kevin T. Roberts, Caroline M. Williams

**Affiliations:** Department of Integrative Biology, University of California, Berkeley, Berkeley, CA 94720, USA

**Keywords:** Climate change, Diapause, Metabolic intensity, Snow cover, Thermal sensitivity, Ecophysiological model

## Abstract

Understanding the energetic consequences of climate change is critical to identifying organismal vulnerabilities, particularly for dormant organisms relying on finite energy budgets. Ecophysiological energy use models predict long-term energy use from metabolic rate, but we do not know the degree to which plasticity in metabolism impacts estimates. We quantified metabolic rate–temperature relationships of dormant willow leaf beetles (*Chrysomela aeneicollis*) monthly from February to May under constant and variable acclimation treatments. Metabolic rate increased as diapause progressed, and acclimation to variable conditions altered both metabolic intensity and thermal sensitivity. However, incorporating these two types of metabolic plasticity into energy use models did not improve energy use estimates, validated by empirical measurements of energy stores. While metabolic rate–temperature relationships are plastic during winter, the magnitude of inter-individual variability in energy stores overshadows the effects of incorporating plasticity into energy use models, highlighting the importance of within-population variation in energy reserves.

## Abstract (precis)

**Summary:** Metabolic rate–temperature relationships are plastic in dormant willow beetles, changing through time and in response to temperature acclimation. Incorporating plasticity into energy use models does not improve their accuracy.

## INTRODUCTION

Understanding the energetic impact of climate change is an important aspect of global change biology, which can reveal critical patterns of vulnerability that temperature alone cannot detect (Dillon et al., 2010; Fitzpatrick et al., 2019). Ecophysiological energy use models predict energy use by mapping body temperature (often estimated from microclimate temperature) onto energetic expenditure using metabolic rate–temperature relationships (Kearney and Porter, 2020; Sinclair et al., 2016, 2013). However, these models assume that metabolic rate–temperature relationships are static, an assumption that is clearly violated given the large amount of phenotypic plasticity that occurs in response to microclimate variation or developmental change (Sinclair, 2015; Sinclair et al., 2016). The impact of plastic variation in metabolic rate–temperature curves on energy use modelling has not been well explored, which may give rise to misleading energy estimates, or overlook important periods of energetic drain. Here, we examined the importance of incorporating metabolic plasticity into winter energy use models in dormant overwintering insects.

Energy conservation is particularly relevant to insect fitness in winter, when finite energy stores must sustain life and also fuel subsequent reproduction (Hahn and Denlinger, 2011; Sinclair, 2015). To conserve limited energy stores, many insects overwinter in diapause – a programmed dormancy characterized by arrested (or slowed) development and metabolic suppression (Koštál, 2006; Wilsterman et al., 2021). Insect metabolic rate during diapause is a fraction (varying from 10% to 85%) of active metabolic rate of comparable developmental stages (Hahn and Denlinger, 2011; Ragland et al., 2009). Metabolic rate changes dynamically as diapause progresses (Koštál, 2006), reaching a minimum several weeks after onset (Toxopeus et al., 2021), followed by a gradual increase towards the time of diapause termination, usually in spring (Gray et al., 1995; Lester and Irwin, 2012).

Metabolic rate–temperature relationships during winter can be altered in two main ways: (1) by changing the intercept, which represents overall metabolic intensity, or (2) by changing the slope, representing thermal sensitivity (Terblanche et al., 2009). Both metabolic intensity and thermal sensitivity change as a result of phenotypic plasticity, including developmental plasticity (e.g. by proceeding through stages of diapause) and acclimation or acclimatization to winter microclimates (Gray et al., 1995; Lester and Irwin, 2012; Williams et al., 2012b). Variable winter microclimates lead to decreased thermal sensitivity, while warmer winter microclimates lead to decreased metabolic intensity, both leading to energetic savings in energetically demanding environments (Sgolastra et al., 2010; Williams et al., 2015, 2012b). Developing a general method to incorporate these widespread patterns of phenotypic plasticity into energy use models may improve our ability to accurately predict winter energy use.

*Chrysomela aeneicollis* (Schaeffer 1928) populations in the Sierra Nevada mountains are an important model system for understanding the physiological and genetic basis of responses to climate change (Dahlhoff et al., 2019, 2008; Rank and Dahlhoff, 2002). Freeze-tolerant adults overwinter in diapause in the soil, often beneath snow, for up to 8 months of their 1 year life cycle (Boychuk et al., 2015). Snow cover modifies the thermal environment of soil by buffering from cold air temperatures (Pauli et al., 2013), providing a relatively warm and stable thermal environment for overwintering insects that can impact energetics and fitness (Irwin and Lee, 2003). Ecophysiological energy use models suggest that energetic costs of winter decrease across elevation, and that snowy years are more energetically demanding than dry years as a result of longer winter periods (Roberts et al., 2021), but previous energy use models used a single metabolic rate–temperature curve with no plasticity taken into account. In this study, we addressed two main objectives: first, we empirically tested how metabolic rate–temperature relationships of *C. aeneicollis* change throughout dormancy and in response to thermal acclimation to ecologically relevant microclimates associated with winter snow. Second, we used ecophysiological models to assess how these modifications will impact energy use estimates, by predicting energy use using different metabolic rate–temperature curves. We then compared these predictions with actual energy use derived from empirical measurements of energy reserves.

## MATERIALS AND METHODS

### Beetle collection and acclimation treatments

Beetles were collected in August 2018 from Mosquito Flat (3067 m) in the Rock Creek Drainage (37°26′25.8″N, 118°44′46.0″W) in the Eastern Sierra Nevada Mountains in CA, USA. Beetles were then housed in incubators (MIR-154-PA incubators, Panasonic Scientific, Wood Dale, IL, USA) in the laboratory at a 20/4°C 12 h:12 h day:night cycle until they entered dormancy, following protocols in Roberts et al. (2021). Once beetles entered dormancy (determined by cessation of feeding in the presence of food; between 21 September and 11 October 2018), they were housed in 50 ml conical tubes filled to around 40% with coconut husk substrate, and moved to an incubator held at 1°C in constant darkness. On 1 November 2018, the beetles (*N*=120) were haphazardly divided between a constant and variable acclimation treatment in separate incubators under constant darkness. The constant treatment simulated conditions beneath the snow (constant 1°C), while the variable treatment simulated uncovered ground (−2.5/−1/0/−1°C in a 6/6/6/6 h cycle). Temperature regimes were selected to mimic late-winter conditions, based on temperature measurements from plots subject to an experimental snow manipulation (Roberts et al., 2021). Beetles were kept in these acclimation treatments, with moisture being added monthly to keep the substrate from drying (around 2–5 ml per month), until their respiration rates were measured.

### Measuring respiration rates

To capture changes in the metabolic rate–temperature relationship through the end of dormancy, oxygen consumption rate (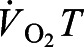) was measured using a Sable Systems FoxBox respirometer (Sable Systems International, North Las Vegas, NV, USA) stop-flow respirometry system once a month from February to May, following protocols from Roberts et al. (2021). We chose these time points to best reflect metabolic rate through the transition out of the coldest portion of dormancy. Briefly, at each time point a subset of 15 beetles from each acclimation treatment (constant and variable) were individually placed into a 10 ml syringe and flushed with CO_2_- and H_2_O-free air, generated using a Drierite–Ascarite–-Drierite column, and then incubated at −1, 4 or 9°C for 48 h, or 20°C for 24 h before oxygen consumption was measured. The order of temperature exposures for the three lowest temperatures was randomized, but always finished with the 20°C measurement to prevent any downstream impacts of prolonged exposure to warm temperatures on respiration rate. Beetles were given a minimum of 48 h to recover between each measurement, during which time they were returned to their acclimation treatment following Lake et al. (2013). All measurements were taken at room temperature, with each syringe being removed from a given incubator within 20 s of the measurement being taken. 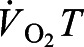 was calculated from oxygen measurements by integration of the area under the curve in Expedata (Sable Systems International), then corrected for air left in the syringe and divided by time spent in the syringe [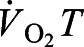
=(5 ml/3 ml)/time] (Lighton, 2018). After each beetle was measured at all temperatures, beetles were weighed then frozen for biochemical analysis.

### Fitting metabolic rate–temperature curves

All analyses were performed in R 4.0.2 (http://www.R-project.org/) unless otherwise specified. 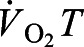 was log-transformed (log_e_) prior to analyses to approximate a linear relationship with temperature. Metabolic rate–temperature curves were fitted to the data using a linear mixed effect model with the lmer function from the lme4 package. We used a Kenward–Roger approximation in the lmerTest package to approximate degrees of freedom and *P*-values (Bates et al., 2015; Kuznetsova et al., 2017). Model selection was done by starting with a full factorial model and eliminating terms using a backwards reduced Kenward–Roger degrees of freedom method with the lmerTest package, which eliminates fixed effects via marginal contrasts of degrees of freedom (Table S1). The initial model included 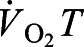 as the dependent variable and month, measurement temperature and acclimation treatment as fixed effects, and mass as a covariate, along with their interaction terms. As 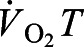 measurements were repeated measures, beetle identity was included in the model as a random effect. The final model included month, measurement temperature, acclimation treatment, mass and the interaction of acclimation treatment and temperature as fixed effects, and individual as a random effect (Table S2). Once the best fit model was chosen and significant terms were identified, we then made models that identified the slope and intercepts of monthly values and treatment independently. Slopes and intercepts were extracted and used to approximate thermal sensitivity and metabolic intensity in energy use models.

### Ecophysiological energy use models

We compared the performance of three ecophysiological energy use models. The first model was from Roberts et al. (2021), based on a single metabolic rate–temperature curve. We refer to this as the ‘base’ model, and all models were modified from this starting model (Eqn 1):
(1)

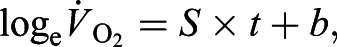
where *S* indicates thermal sensitivity, *t* indicates temperature and *b* represents metabolic intensity. The second model incorporates a stepwise monthly increase in the intercept, simulating the gradual increase in metabolic intensity that we documented between months in the respiration rate measurements, with an intercept of −1.952 and a slope of 0.173. We determined the rate of change in the intercept by fitting a linear regression to the intercept of the metabolic rate–temperature relationship as a function of month (February–May), and used the slope of this regression as a scaling factor in the static model. We refer to this as the ‘dynamic’ model (Eqn 2):
(2)

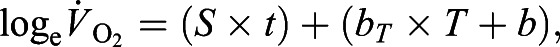
where *T* is month of measurement, and *b_T_* is the monthly rate of change in metabolic intensity. The third model incorporated the impact of plasticity in response to acclimation treatment (see Results). The empirical metabolic rate–temperature relationship from Roberts et al. (2021) was measured in beetles maintained under constant acclimation conditions. Using the base model as the basis, we modified both *S* and *b* by the amount that they were modified in the variable compared with constant acclimation treatment in the present study. We refer to this as the ‘acclimation’ model, and it was used only in predictions of energy use in the variable acclimation treatment (Eqn 3):
(3)

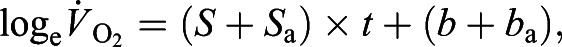
where *S*_a_ refers to the difference in thermal sensitivity and *b*_a_ the difference in metabolic intensity between the two acclimation treatments.

Hourly oxygen consumption throughout the winter was calculated using Eqns 1–3, using temperatures (*t*) from the constant and variable acclimation treatments. Monthly energy use was calculated by summing hourly oxygen consumption, and converted to lipid consumed assuming 2 l oxygen per 1 g lipid metabolized (Schmidt-Nielsen, 1997), giving rise to monthly lipid use estimates under each model, for each acclimation treatment.

### Empirically validating model estimates

We quantified lipid (triacylglyceride) stores of beetles (15 per month per treatment) using thin-layer chromatography coupled to a flame ionization detector (TLC-FID, Iatroscan MK-6s TLC-FID Analyzer, Shell-USA, Spotsylvania, VA, USA), with cholesterol as an internal standard (Roberts et al., 2021; Williams et al., 2011). We tested whether lipid stores changed through time by ANOVA in which month, acclimation and their interaction were included as independent variables and lipid stores as the dependent variable.

To validate model predictions of lipid use against these empirical measurements of lipid stores, we subtracted the monthly lipid use estimates for each model (Eqns 1–3) from the average February lipid stores for constant and variable acclimation separately, giving us predicted monthly lipid stores. We expressed cumulative model errors (over the entire period of February to May) as the absolute difference between lipid stores of individual beetles in May and predicted lipid stores in May under each model, separately for constant and variable incubators. Model accuracy was compared between models using ANOVA with absolute errors as the response variable and model as the predictor, separately for each acclimation treatment.

## RESULTS AND DISCUSSION

### Plasticity in the metabolic rate–temperature relationship

**Fig. 1.**
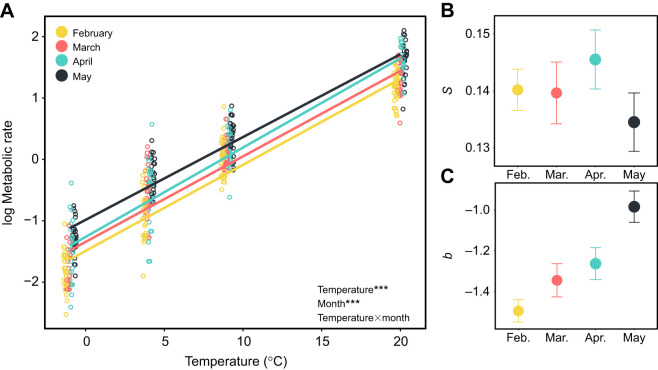
**Developmental plasticity in metabolic rate–temperature relationships in *Chrysomela aeneicollis*.** (A) Metabolic rate (estimated using rate of oxygen consumption, 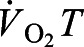; μl O_2_ h^−1^) as a function of temperature for beetles measured in February, March, April and May, as indicated. (B) Thermal sensitivity (*S*; slope) and (C) metabolic intensity (*b*; intercept) of the metabolic rate–temperature relationships in A. Data shown were combined from variable and constant treatments as the effect of time did not differ by treatment. Error bars in B and C denote standard errors on the parameter estimates. Significance: ***P<0.001 from a linear fixed effects model.

Beetle metabolic rate–temperature relationships changed through time, and in response to thermal acclimation. Beetle metabolic rate increased between February and May, resulting in a gradual increase in metabolic intensity with no corresponding change in thermal sensitivity (*F*_3,108_=32.27, *P*<0.0001; Fig. 1; Table S2). A similar increase of metabolic intensity during diapause development has been observed in pine beetles (Lester and Irwin, 2012), gypsy moths (Gray et al., 1995) and solitary bees (Sgolastra et al., 2010) but not goldenrod gall flies (Irwin et al., 2001), and occurs during the transition from diapause to post-diapause quiescence (Hahn and Denlinger, 2011; Lester and Irwin, 2012; Ragland et al., 2009). We thus consider it likely that the increase in metabolic rate through time results from developmental plasticity due to diapause progression (Koštál, 2006), although we cannot rule out some influence of other factors that change through time over winter (e.g. long-term low temperature acclimation). Paralleling our results of no change in thermal sensitivity, thermal sensitivity remains consistent throughout diapause in gypsy moth eggs (Gray et al., 1995), but increases towards the end of diapause in fall webworm pupae (Williams et al., 2015), suggesting that changes in thermal sensitivity throughout diapause may be less important and consistent than changes in overall metabolic intensity.

**Fig. 2.**
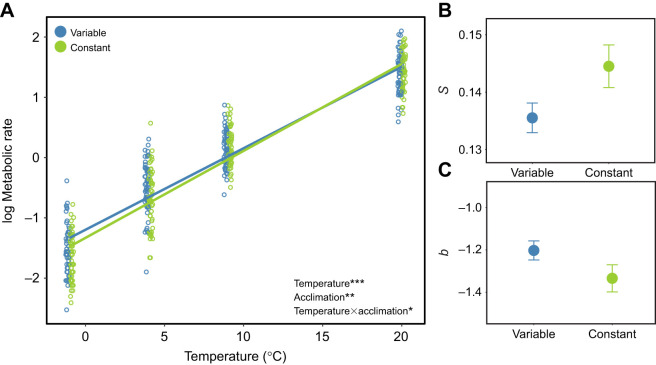
**Plasticity in metabolic rate–temperature relationships as a result of thermal acclimation in *C. aeneicollis*.** (A) Metabolic rate data (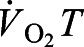; μl O_2_ h^−1^) from beetles that overwintered in variable (temperatures between −2.5 and 0°C daily) and constant (1°C) acclimation treatments, with monthly measurements pooled. (B) Thermal sensitivity (*S*; slope) and (C) metabolic intensity (*b*; intercept) of the metabolic rate–temperature relationships in A. Error bars in B and C denote standard errors on the parameter estimates. Significance: ***P<0.001, **P<0.01, *P<0.05 from a linear fixed effects model.

Beetles acclimated to variable temperatures had higher metabolic intensity than beetles acclimated to constant conditions (*F*_1,237_=9.74, *P*=0.0020; Fig. 2C; Table S2), and lower thermal sensitivity (*F*_1,332_=5.48, *P*=0.0198; Fig. 2B; Table S2). Variable winter temperatures also resulted in lower thermal sensitivity in overwintering larval Lepidoptera (Williams et al., 2012b). Thermal regimes approximated soil temperatures below snow (constant) or with snow removed (variable) at a high elevation site in the Eastern Sierra Nevada mountains (Roberts et al., 2021), so it is likely that natural variation in snow cover would elicit a plastic response in metabolic rate of these beetles.

### Impact of metabolic plasticity on long-term energy use

**Fig. 3.**
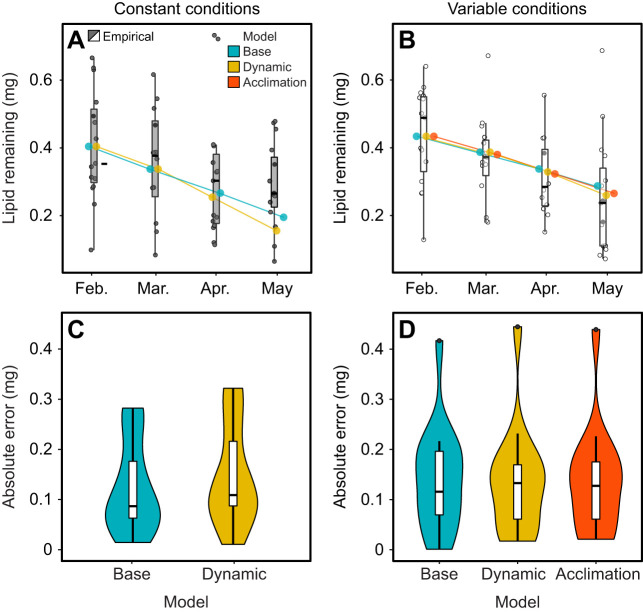
**Energy use model estimates are not improved by incorporating metabolic rate plasticity when compared with empirical lipid measurements.** Data shown are energy use estimates from energy use models incorporating no plasticity (‘base’ model), developmental plasticity (‘dynamic’ model) and acclimation plasticity (‘acclimation’ model) overlain on empirical lipid quantifications (gray) for *C. aeneicollis* beetles that overwintered in constant (A) or variable (B) acclimation treatments. Estimates from the base model are provided for both constant and variable acclimation treatments to allow comparison of model estimates in the same environment. (C) Errors between model predictions of lipid stores compared with empirically measured lipid stores of beetles in May (absolute value of model output subtracted from individual empirical measurement) in constant conditions. (D) Errors between model predictions of lipid stores compared with empirically measured lipid stores of beetles in May in variable conditions. For all panels, boxplots show median, upper and lower 25% quartile, and range, with raw data overplotted as circles in A and B.

Beetle lipid stores decreased linearly through time (*F*_3,102_=2.87, *P*=0.040; Table S3), and did not differ between constant and variable acclimation treatments (*F*_1,104_=0.008, *P*=0.93, Fig. 3A,B; Table S3). All models predicted energy use accurately, with estimates falling within the 25th to 75th percentile of empirically measured beetle lipid stores, with the exception of the constant acclimation treatment in May (Fig. 3A). This illustrates that the base model used in Roberts et al. (2021) predicts energy use accurately under laboratory, as well as field, conditions. Neither the dynamic model nor the acclimation model, incorporating population-level plasticity in response to acclimation, improved the accuracy of model estimates of whole winter energy use relative to the base model used in Roberts et al. (2021) (*F*_2,84_=0.33, *P*=0.72; Fig. 3C,D; Table S4). Thus, incorporating population-level plasticity in metabolic rate (as a result of acclimation or developmental plasticity due to diapause progression) did not improve population-level estimates of energy use from February to May. This suggests that ecologically relevant magnitudes of plasticity will not compromise population-level estimates of energy use.

The mismatch between predictions of the dynamic model and empirical energy reserve estimates was striking. The dynamic model predicted accelerating rates of energy use throughout diapause, as a result of the observed increase in metabolic intensity, but we did not see any increase in rates of empirical lipid depletion throughout diapause (if anything, rates of lipid depletion slowed as diapause progressed, particularly in the constant conditions). This may have occurred as a consequence of a physiological shift that decoupled oxygen consumption and lipid use (e.g. switching to an alternative fuel source). This underlines the hazards in using thermal performance curves measured over short time scales to estimate long-term responses (Kingsolver and Buckley, 2017; Sinclair et al., 2016). It is possible that our ability to detect differences in energy use that correlate with changes in the metabolic rate–temperature curves would be improved by an individual-based approach, which would require measuring metabolic rate and energy reserves repeatedly on individual organisms (e.g. by qNMR; Guglielmo et al., 2011), but these experiments are technically challenging with small insects at present.

The magnitude of plasticity that we observed between months or acclimation treatments was dwarfed by the magnitude of variation in individual energy stores. The largest magnitude of plasticity in our experiment was developmental (presumably due to diapause progression) and, in the constant acclimation treatment, the difference in predicted energy use resulting from developmental plasticity was 0.04 mg (base versus dynamic model; Fig. 3A). However, there was large individual variation in observed lipid stores (s.d.=0.15 mg), swamping the magnitude of plasticity. Based on observed levels of variability, the magnitude of difference between model estimates would have to exceed ∼0.18 mg of lipid in order to detect a statistically significant effect (power analysis; power=0.8, *n*=15, α=0.05). To detect the observed predicted difference in energy use between the base and dynamic model in the constant environment (0.04 mg lipid), we would need a sample size of 112 per treatment (or 7.5 times what was used in this study). The mean (±s.d.) mass of beetles was 15.7±3.9 mg, and mean lipid content was 0.42±0.21 mg, making the standard deviation 25% of the mean. This is well within the 20–38% variance seen in across four other species of overwintering beetles (Lehmann et al., 2020; Watanabe and Tanaka, 2000) and the 20–41% variance in lepidopterans (Marshall and Sinclair, 2012; Williams et al., 2015, 2012b, 2011), indicating that individual variability is worth considering across insect taxa. The large degree of variation among individuals in lipid stores is likely to have more profound impacts on energetic stress in winter than the magnitude of plasticity that we observed. Individual lipid stores are vital in determining an organism's fasting endurance in winter (Trondrud et al., 2021), and may provide an interesting avenue for future research.

The degree of metabolic plasticity that we observed may be more impactful in natural thermal environments, which exhibit greater variation in winter energetic demands than did our two acclimation treatments (i.e. as a result of a wider range of temperatures or longer overwintering periods). Energy use estimates from models incorporating developmental plasticity (increase in metabolic intensity) would be particularly sensitive to warm temperatures because of the non-linear relationship of metabolic rate with temperature (Jensen's inequality; Ruel and Ayres, 1999) combined with the observed increase in metabolic intensity. This could have large impacts on overwinter energy use, where warm temperatures at the end of winter can heavily influence energy expenditure (Roberts et al., 2021). Adding the developmental plasticity to model estimates of winter energy use from Roberts et al. (2021) led to an average increase of 24.3% or 0.149 mg lipid, and when including thermal acclimation there was an 11.0% or 0.064 mg increase. As an ∼0.18 mg lipid difference is needed to detect an effect given intraspecific variability in lipid stores, acclimation plasticity will have negligible impacts, but developmental plasticity may come close to causing detectable differences in energy use in more ecologically relevant thermal regimes.

The energetic costs of winter are a critical and understudied aspect of understanding biotic responses to climate change (Fitzpatrick et al., 2020; Sinclair, 2015; Williams et al., 2012a). Metabolic rate–temperature relationships in dormancy are plastic, changing as a result of developmental plasticity and thermal acclimation. We developed a novel method to incorporate these types of plasticity into energy use models in a generalizable way, but this did not improve our model estimates relative to empirical lipid quantifications. We instead found that individual variation in lipid stores far overshadowed the effects of plasticity on energy use. This suggests that estimating winter energy use based on a single metabolic rate–temperature relationship may give us reasonable energy use estimates, and that individual variability in lipid stores is a critical component that is often overlooked, but may provide valuable insight that will help us to understand the energetic impacts of environmental change.

## Supplementary Material

Supplementary information

## References

[JEB243422C1] Bates, D., Mächler, M., Bolker, B. and Walker, S. (2015). Fitting linear mixed-effects models using lme4. *J. Stat. Software* 67, 1-48. 10.18637/jss.v067.i01

[JEB243422C2] Boychuk, E. C., Smiley, J. T., Dahlhoff, E. P., Bernards, M. A., Rank, N. E. and Sinclair, B. J. (2015). Cold tolerance of the montane Sierra leaf beetle, Chrysomela aeneicollis. *J. Insect Physiol.* 81, 157-166. 10.1016/j.jinsphys.2015.07.015 26231921

[JEB243422C3] Dahlhoff, E. P., Fearnley, S. L., Bruce, D. A., Gibbs, A. G., Stoneking, R., McMillan, D. M., Deiner, K., Smiley, J. T. and Rank, N. E. (2008). Effects of temperature on physiology and reproductive success of a montane leaf beetle: implications for persistence of native populations enduring climate change. *Physiol. Biochem. Zool.* 81, 718-732. 10.1086/590165 18956974

[JEB243422C4] Dahlhoff, E. P., Dahlhoff, V. C., Grainger, C. A., Zavala, N. A., Otepola-Bello, D., Sargent, B. A., Roberts, K. T., Heidl, S. J., Smiley, J. T. and Rank, N. E. (2019). Getting chased up the mountain: high elevation may limit performance and fitness characters in a montane insect. *Funct. Ecol.* 33, 809-818. 10.1111/1365-2435.13286

[JEB243422C5] Dillon, M. E., Wang, G. and Huey, R. B. (2010). Global metabolic impacts of recent climate warming. *Nature* 467, 704-706. 10.1038/nature09407 20930843

[JEB243422C6] Fitzpatrick, M. J., Zuckerberg, B., Pauli, J. N., Kearney, M. R., Thompson, K. L., Werner, L. C. and Porter, W. P. (2019). Modeling the distribution of niche space and risk for a freeze-tolerant ectotherm, Lithobates sylvaticus. *Ecosphere* 10, e02788. 10.1002/ecs2.2788

[JEB243422C7] Fitzpatrick, M. J., Porter, W. P., Pauli, J. N., Kearney, M. R., Notaro, M. and Zuckerberg, B. (2020). Future winters present a complex energetic landscape of decreased costs and reduced risk for a freeze-tolerant amphibian, the Wood Frog (Lithobates sylvaticus). *Glob. Change Biol.* 26, 6350-6362. 10.1111/gcb.15321 32871618

[JEB243422C8] Gray, D. R., Ravlin, F. W., Régnière, J. and Logan, J. A. (1995). Further advances toward a model of gypsy moth (Lymantria dispar (L.)) egg phenology: respiration rates and thermal responsiveness during diapause, and age-dependent developmental rates in postdiapause. *J. Insect Physiol.* 41, 247-256. 10.1016/0022-1910(94)00102-M

[JEB243422C9] Guglielmo, C. G., McGuire, L. P., Gerson, A. R. and Seewagen, C. L. (2011). Simple, rapid, and non-invasive measurement of fat, lean, and total water masses of live birds using quantitative magnetic resonance. *J. Ornithol.* 152, 75. 10.1007/s10336-011-0724-z

[JEB243422C10] Hahn, D. A. and Denlinger, D. L. (2011). Energetics of insect diapause. *Annu. Rev. Entomol.* 56, 103-121. 10.1146/annurev-ento-112408-085436 20690828

[JEB243422C11] Irwin, J. T. and Lee, R. E. (2003). Cold winter microenvironments conserve energy and improve overwintering survival and potential fecundity of the goldenrod gall fly, Eurosta solidaginis. *Oikos* 100, 71-78. 10.1034/j.1600-0706.2003.11738.x

[JEB243422C12] Irwin, J. T., Bennett, V. A. and Lee, R. Jr. (2001). Diapause development in frozen larvae of the goldenrod gall fly, Eurosta solidaginis Fitch (Diptera: Tephritidae). *J. Comp. Physiol. B* 171, 181-188. 10.1007/s003600000154 11352100

[JEB243422C13] Kearney, M. R. and Porter, W. P. (2020). NicheMapR–an R package for biophysical modelling: the ectotherm and Dynamic Energy Budget models. *Ecography* 43, 85-96. 10.1111/ecog.04680

[JEB243422C14] Kingsolver, J. G. and Buckley, L. B. (2017). Quantifying thermal extremes and biological variation to predict evolutionary responses to changing climate. *Philos. Trans. R. Soc. B Biol. Sci.* 372, 20160147. 10.1098/rstb.2016.0147 PMC5434097 28483862

[JEB243422C15] Koštál, V. (2006). Eco-physiological phases of insect diapause. *J. Insect Physiol.* 52, 113-127. 10.1016/j.jinsphys.2005.09.008 16332347

[JEB243422C16] Kuznetsova, A., Brockhoff, P. B. and Christensen, R. H. B. (2017). lmerTest Package: tests in linear mixed effects models. *J. Stat. Software* 82, 26. 10.18637/jss.v082.i13

[JEB243422C17] Lake, S. L., MacMillan, H. A., Williams, C. M. and Sinclair, B. J. (2013). Static and dynamic approaches yield similar estimates of the thermal sensitivity of insect metabolism. *J. Insect Physiol.* 59, 761-766. 10.1016/j.jinsphys.2013.04.010 23665211

[JEB243422C18] Lehmann, P., Westberg, M., Tang, P., Lindström, L. and Käkelä, R. (2020). The diapause lipidomes of three closely related beetle species reveal mechanisms for tolerating energetic and cold stress in high-latitude seasonal environments. *Front. Physiol.* 11, 1219. 10.3389/fphys.2020.576617 PMC7546402 33101058

[JEB243422C19] Lester, J. D. and Irwin, J. T. (2012). Metabolism and cold tolerance of overwintering adult mountain pine beetles (Dendroctonus ponderosae): Evidence of facultative diapause? *J. Insect Physiol.* 58, 808-815. 10.1016/j.jinsphys.2012.03.003 22426083

[JEB243422C20] Lighton, J. R. (2018). *Measuring Metabolic Rates: A Manual for Scientists*. Oxford University Press.

[JEB243422C21] Marshall, K. E. and Sinclair, B. J. (2012). Threshold temperatures mediate the impact of reduced snow cover on overwintering freeze-tolerant caterpillars. *Naturwissenschaften* 99, 33-41. 10.1007/s00114-011-0866-0 22139093

[JEB243422C22] Pauli, J. N., Zuckerberg, B., Whiteman, J. P. and Porter, W. (2013). The subnivium: a deteriorating seasonal refugium. *Front. Ecol. Environ.* 11, 260-267. 10.1890/120222

[JEB243422C23] Ragland, G. J., Fuller, J., Feder, J. L. and Hahn, D. A. (2009). Biphasic metabolic rate trajectory of pupal diapause termination and post-diapause development in a tephritid fly. *J. Insect Physiol.* 55, 344-350. 10.1016/j.jinsphys.2008.12.013 19200436

[JEB243422C24] Rank, N. E. and Dahlhoff, E. P. (2002). Allele frequency shifts in response to climate change and physiological consequences of allozyme variation in a montane insect. *Evolution* 56, 2278-2289. 10.1111/j.0014-3820.2002.tb00151.x 12487357

[JEB243422C25] Roberts, K. and Williams, C. M. (2022). The impact of metabolic plasticity on winter energy use models. *Dryad, Dataset*. https://doi.org/10.6078/D1N13N 10.1242/jeb.243422 PMC8920032 35098313

[JEB243422C26] Roberts, K. T., Rank, N. E., Dahlhoff, E. P., Stillman, J. H. and Williams, C. M. (2021). Snow modulates winter energy use and cold exposure across an elevation gradient in a montane ectotherm. *Glob. Change Biol.* 27, 6103-6116. 10.1111/gcb.15912 34601792

[JEB243422C27] Ruel, J. J. and Ayres, M. P. (1999). Jensen's inequality predicts effects of environmental variation. *Trends Ecol. Evol.* 14, 361-366. 10.1016/S0169-5347(99)01664-X 10441312

[JEB243422C28] Schmidt-Nielsen, K. (1997). *Animal Physiology: Adaptation and Environment*. Cambridge University Press.

[JEB243422C29] Sgolastra, F., Bosch, J., Molowny-Horas, R., Maini, S. and Kemp, W. P. (2010). Effect of temperature regime on diapause intensity in an adult-wintering Hymenopteran with obligate diapause. *J. Insect Physiol.* 56, 185-194. 10.1016/j.jinsphys.2009.10.001 19837077

[JEB243422C30] Sinclair, B. J. (2015). Linking energetics and overwintering in temperate insects. *J. Therm. Biol.* 54, 5-11. 10.1016/j.jtherbio.2014.07.007 26615721

[JEB243422C31] Sinclair, B. J., Stinziano, J. R., Williams, C. M., MacMillan, H. A., Marshall, K. E. and Storey, K. B. (2013). Real-time measurement of metabolic rate during freezing and thawing of the wood frog, Rana sylvatica: implications for overwinter energy use. *J. Exp. Biol.* 216, 292-302. 10.1242/jeb.076331 23255194

[JEB243422C32] Sinclair, B. J., Marshall, K. E., Sewell, M. A., Levesque, D. L., Willett, C. S., Slotsbo, S., Dong, Y., Harley, C. D. G., Marshall, D. J. and Helmuth, B. S. (2016). Can we predict ectotherm responses to climate change using thermal performance curves and body temperatures? *Ecol. Lett.* 19, 1372-1385. 10.1111/ele.12686 27667778

[JEB243422C33] Terblanche, J. S., Clusella-Trullas, S., Deere, J. A., Van Vuuren, B. J. and Chown, S. L. (2009). Directional evolution of the slope of the metabolic rate–temperature relationship is correlated with climate. *Physiol. Biochem. Zool.* 82, 495-503. 10.1086/605361 19624273

[JEB243422C34] Toxopeus, J., Gadey, L., Andaloori, L., Sanaei, M. and Ragland, G. J. (2021). Costs of averting or prematurely terminating diapause associated with slow decline of metabolic rates at low temperature. *Comp. Biochem. Physiol. A Mol. Integr. Physiol.* 255, 110920. 10.1016/j.cbpa.2021.110920 33582264

[JEB243422C35] Trondrud, L. M., Pigeon, G., Król, E., Albon, S., Evans, A. L., Arnold, W., Hambly, C., Irvine, R. J., Ropstad, E., Stien, A. et al. (2021). Fat storage influences fasting endurance more than body size in an ungulate. *Funct. Ecol.* 35, 1470-1480. 10.1111/1365-2435.13816

[JEB243422C36] Watanabe, M., Tanaka, K. (2000). Hormonal control of diapause and overwintering traits in a leaf beetle, Aulacophora nigripennis. *Physiol. Entomol.* 25, 337-345. 10.1046/j.1365-3032.2000.00202.x

[JEB243422C37] Williams, C. M., Thomas, R. H., MacMillan, H. A., Marshall, K. E. and Sinclair, B. J. (2011). Triacylglyceride measurement in small quantities of homogenised insect tissue: comparisons and caveats. *J. Insect Physiol.* 57, 1602-1613. 10.1016/j.jinsphys.2011.08.008 21878339

[JEB243422C38] Williams, C. M., Hellmann, J. and Sinclair, B. J. (2012a). Lepidopteran species differ in susceptibility to winter warming. *Clim. Res.* 53, 119-130. 10.3354/cr01100

[JEB243422C39] Williams, C. M., Marshall, K. E., MacMillan, H. A., Dzurisin, J. D. K., Hellmann, J. J. and Sinclair, B. J. (2012b). Thermal variability increases the impact of autumnal warming and drives metabolic depression in an overwintering butterfly. *PLoS ONE* 7, e34470. 10.1371/journal.pone.0034470 22479634 PMC3316672

[JEB243422C40] Williams, C. M., Chick, W. D. and Sinclair, B. J. (2015). A cross–seasonal perspective on local adaptation: metabolic plasticity mediates responses to winter in a thermal–generalist moth. *Funct. Ecol.* 29, 549-561. 10.1111/1365-2435.12360

[JEB243422C41] Wilsterman, K., Ballinger, M. A. and Williams, C. M. (2021). A unifying, eco–physiological framework for animal dormancy. *Funct. Ecol.* 35, 11-31. 10.1111/1365-2435.13718

