# Supplementary material for: The impact of metabolic plasticity on winter energy use models
Source: J Exp Biol. 2022 Feb 25;225(4):jeb243422. doi: 10.1242/jeb.243422 (PMC8920032; doi:10.1242/jeb.243422)
Supplement: Supplementary information [file jexbio-225-243422-s1.pdf]

Table S1. Model selection process for fitting V O_2 using a backwards reduced Kenward-Roger degrees of freedom method with the lmerTest package in R. The top three interaction terms were dropped based on F value reduction, leaving the final model of $\dot{V}O_2$ as a dependent variable and month, acclimation, temperature, and the interaction of temperature and acclimation as fixed effects and individual as a random effect.

	Eliminated	SumSq	MeanSq	NumDF	DenDF	F value	p-value
Temp:Acclim:Month	1	0.338	0.1127	3	323.75	1.2143	0.3045
Acclim:Month	2	0.2484	0.0828	3	102.35	0.8916	0.4482
Temp:Month	3	0.4099	0.1366	3	326.51	1.4708	0.2223
Month	0	9.0374	3.0125	3	105.61	32.2718	<0.0001
Mass	0	3.8236	3.8236	1	107.84	40.9613	<0.0001
Temp:Acclimation	0	0.5118	0.5118	1	329.28	5.4832	0.0198

Table S2. Linear mixed effect model results in an analysis of deviance table from type iii Walf F tests with Kenward-Roger degrees of freedom.

	F	Df	Df.res	p-value
(Intercept)	351.4558	1	119.9	<0.0001
Temperature	2721.3077	1	330.63	<0.0001
Acclimation	9.7375	1	236.93	0.002029
Month	32.2687	3	108.28	<0.0001
Mass	40.9553	1	110.54	<0.0001
Temp:Acclimation	5.4822	1	331.93	0.019802

Table S3. ANOVA model results for lipid from each individual used for respirometry. Lipid data are triacylglyceride abundance quantified via TLC-FID (thin layer chromatography with flame ionization detector).

	SumSq	Df	F value	p-value
(Intercept)	3.03246	1	113.4859	<0.0001
Month	0.22967	3	2.865	0.04015
Acclimation	0.00021	1	0.0078	0.92966
Month:Acclimation	0.00388	3	0.0484	0.98584
Residuals	2.83243	106		

Table S4. ANOVA results from comparing error from model estimates of May lipid levels per model.

	SumSq	Df	F value	p-value
(Intercept)	0.02668	1	1.0117	0.3173
Model	0.01774	2	0.3364	0.7153
Residuals	2.29449	87		
